# Single-Agent Thalidomide for Treatment of Malignant Paraganglioma of the Organ of Zuckerkandl

**DOI:** 10.1155/2019/7185973

**Published:** 2019-09-05

**Authors:** Mackenzie L. Shindorf, Prabir K. Chaudhuri

**Affiliations:** University of Toledo Medical Center, 3000 Arlington Drive, Toledo, OH 43614, USA

## Abstract

The standard of care for benign pheochromocytomas and paragangliomas is surgical resection; however, there is no definitive curative or standard therapy for the treatment of malignant pheochromocytomas or paragangliomas. Current therapeutic options include surgical resection, chemotherapy (CVD therapy), MIBG-radiotherapy, somatostatin analogues, and combination targeted therapies such as temozolomide and thalidomide. Although some patients demonstrate short-lived responses to these various therapies, there has been no statistically significant survival benefit with any of the current regimens and therefore the aim is palliation and symptom control. Here, we present a 38-year-old man found to have an unresectable metastatic paraganglioma located in the Organ of Zuckerkandl who has been treated with single-agent thalidomide for over 15 years with minimal growth and stabilization of metastatic lesions. The management of our patient with single-agent therapy for this extended period of time challenges the efficacy and role of chemotherapy and other accepted therapies for malignant pheochromocytoma and paraganglioma.

## 1. Introduction

Pheochromocytomas are rare catecholamine-secreting tumors, the majority of which are benign and located within the adrenal medulla. Approximately 10% of these tumors are malignant. According to the World Health Organization, malignancy in a pheochromocytoma is defined by presence of regional or distant metastases associated with the primary pheochromocytoma [1–5]. There are no identifiable histologic features or biochemical criteria of the primary tumor that will differentiate a benign from a malignant lesion. Risk factors of malignancy include size >5 cm, extra-adrenal locations, SDH gene mutations, and local invasiveness [1–5].

Extra-adrenal pheochromocytomas, or paragangliomas, have a much higher probability of being malignant [1, 2, 4, 5]. Approximately 10–20% of pheochromocytomas are found in extra-adrenal locations along the sympathetic or parasympathetic chains, most commonly in the Organ of Zuckerkandl, a collection of chromaffin tissue near the inferior mesenteric artery and aortic bifurcation [2, 4, 5].

Standard treatment for benign pheochromocytomas and paragangliomas is surgical resection. There is, however, no current definitive curative or standard therapy for malignant pheochromocytomas or paragangliomas partly due to the rarity of these tumors. Current therapeutic options include attempts at surgical resection; chemotherapy consisting of cyclophosphamide, vincristine, and dacarbazine (CVD); MIBG-radiotherapy; somatostatin analogues; and new antineoplastic therapies including temozolomide and thalidomide [2–5]. These treatments are aimed primarily at palliation and symptom control as there has been no statistically significant survival benefit with any of these therapies although some patients demonstrate short-lived responses.

Despite multiple treatment options, the 5-year mortality rate in patients with malignant pheochromocytomas and paragangliomas is greater than 50% in some reports [5]; however, a recently published retrospective review reports an 85% 5-year overall survival rate [6]. Here, we present a case of a large metastatic paraganglioma of the Organ of Zuckerkandl managed for greater than 15 years with a single-agent therapy of thalidomide.

## 2. Case Presentation

An otherwise healthy 38-year-old man was found on CT to have a large 20 cm × 9 cm mass near the aortic bifurcation with associated areas of calcifications and low attenuated areas concerning for necrosis. In addition, bilateral lung masses, the largest measuring 2 cm, and multiple liver lesions with the largest measuring 3 cm, consistent with metastases, were identified. The patient underwent a CT-guided biopsy of the abdominal mass at an outside community hospital. Pathology and immunohistochemistry staining of synaptophysin and chromogranin established that the mass was consistent with a paraganglioma of the Organ of Zuckerkandl and the patient was subsequently referred to our center.

Full laboratory workup was performed. Twenty-four-hour urine-free catecholamine evaluation revealed elevated epinephrine and norepinephrine levels (epinephrine: 69 mcg/24 hours (normal: 0–24), norepinephrine: 567 mcg/24 hours (normal 0–140), dopamine: 231 mcg/24 hours (normal 65–610)). Twenty-four-hour metanephrine levels were obtained and demonstrated elevated metanephrine and normetanephrine levels (metanephrine: 545 mcg/24 hours (normal: 35–460), normetanephrine: 2082 mcg/24 hours (normal 110–1050)). SDH gene testing was not performed and DOTATE PET CT imaging was unavailable.

After discussion with the patient, he was started on single-agent thalidomide 200 mcg daily based on the lack of effective chemotherapy. The patient was followed closely with repeat imaging every 3 months. Throughout the initial portion of his treatment he remained asymptomatic; however, as his treatment course progressed, he became increasingly hypertensive which was easily controlled with doxazosin. CT scans demonstrated stability in lung and liver metastases as well as significant regression of the paraganglioma to nearly 50% of the original size. Despite stability of disease, after approximately 18 months of thalidomide treatment, he became increasingly symptomatic with mild palpitations. Subsequently, he underwent an exploratory laparotomy in attempt for surgical resection but intraoperatively the tumor was deemed unresectable due to encasement of the aorta and inferior vena cava. With these operative findings, the patient was continued on thalidomide and continues to be closely followed.

**Figure 1:**
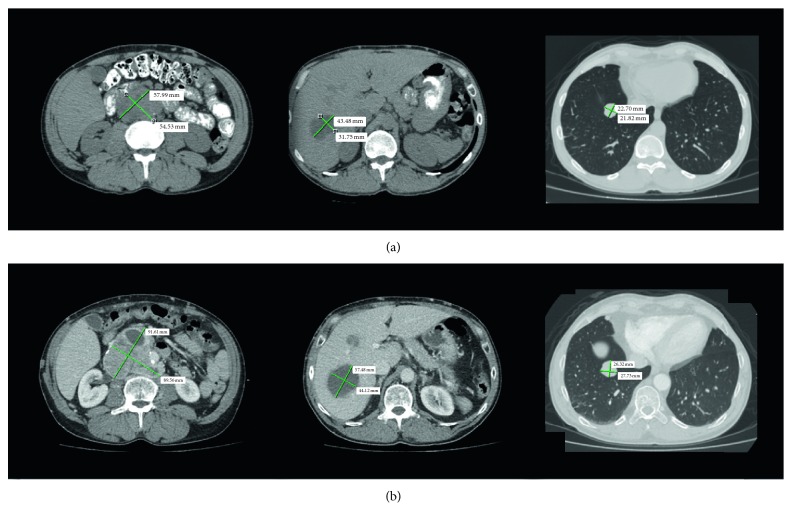
Original imaging from day of diagnosis are not available; however, images 4 years after diagnosis are displayed in (a). The left image demonstrates the primary pheochromocytoma in the Organ of Zuckerkandl. The middle image demonstrates one of the metastatic liver lesions, and the far-right image demonstrates a metastatic lung lesion with their corresponding measurements. Most recent imaging, over 15 years since diagnosis, demonstrates these same lesions with their respective measurements in (b).

At the current time, over 15 years since his initial diagnosis, the patient remains on single-agent thalidomide for tumor control and single antihypertensive therapy without adverse effects. Over the past 15 years, the primary tumor at the Organ of Zuckerkandl and one of the multiple liver metastases have both slowly enlarged by approximately 5 cm and 1 cm, respectively (Figure 1). The remaining liver metastases and lung metastases have continued to be stable. An MIBG scan performed two years ago demonstrates active metastatic lesions in the lungs and liver as well as the primary lesion that demonstrates external compression of the inferior vena cava. 

## 3. Discussion

Here, we presented a patient with a large, malignant paraganglioma of the Organ of Zuckerkandl with metastases to the liver and bilateral lungs treated with single-agent thalidomide for over 15 years. Our patient presented with three known risk factors for a malignant pheochromocytoma: size greater than 5 centimeters, extra-adrenal origin, and local invasiveness.

There is currently no curative or standard therapy regimen for malignant pheochromocytomas or paragangliomas. Most treatments do not show any survival benefit, and surgical resection is not curative [1–5]. The therapeutic options available to patients with metastatic pheochromocytomas and paragangliomas are aimed at symptom control which includes surgical debulking, chemotherapy (consisting of cyclophosphamide, vincristine, and dacarbazine or CVD therapy), radiotherapy, somatostatin analogues, and targeted antineoplastic therapies [2–5]. There are many limitations to these therapies, including the fact that these therapy regimens are lifelong treatments as recurrence occurs after discontinuation of the treatment.

One treatment regimen that is tolerated relatively well is the combination antineoplastic therapy of temozolomide and thalidomide, which is considered a targeted therapy for neuroendocrine tumors [7]. Temozolomide, a cytotoxic alkylating chemotherapy agent, is similar to dacarbazine in its effects on tumors [5, 7]. Dacarbazine is one of the triple agents in the more widely accepted chemotherapy treatment of pheochromocytomas. The only acceptable route of administration for dacarbazine is intravenously. However, the benefit of temozolomide is that oral administration is the preferred route of administration [1–5]. Thalidomide, an antiangiogenesis drug, prevents the formation of new vasculature by inhibiting the effects of vascular endothelial growth factor (VEGF) [8]. The combination of these drugs has demonstrated radiographic and biochemical response in upwards of 33% of patients, yet it does not show any significant survival benefit and is not a curative treatment [7].

Most patients regardless of the therapy administered for treatment of malignant pheochromocytomas or paragangliomas have a poor prognosis with a historical five-year survival rate of less than 50% [5] although newer reports have challenged these data [6]. We present a patient with a malignant paraganglioma who has only been treated with single-agent thalidomide for over 15 years. This patient and treatment regimen elicits multiple questions regarding the current treatment options and effectiveness of these therapies. Primarily, this challenges both the efficacy and role of chemotherapy compared to and in combination with targeted therapies, specifically thalidomide, in the treatment of malignant pheochromocytomas and paragangliomas. Secondarily, it raises questions regarding the biology of the patient's tumor as the majority of patients would have succumbed to their disease at this point despite treatment. The rarity of these tumors makes understanding the natural history of the disease difficult but more information is needed in order to create a standard of care treatment regimen for such patients. Recognizing that this effect was seen in a single patient, further studies are needed to explore the potential for using single-agent thalidomide as a therapeutic option in attempt to standardize the treatment of malignant pheochromocytomas and paragangliomas.

## 4. Conclusions

Malignant pheochromocytomas and paragangliomas are rare and challenging tumors to manage for many reasons as there are no identifiable histological or biochemical differences to predict benign and malignant disease. The determination of benign versus malignant disease lies solely in the presence or absence of local or distant metastatic disease. These tumors are rare enough that the biology of the disease is still being investigated and is not fully understood. Due to this lack of full understanding of the disease, the treatment options for malignant pheochromocytomas or paragangliomas are directed toward symptom control and no standard of care treatment regimen has been elucidated. Here was presented a case of a single patient who after diagnosis of a malignant paraganglioma of the Organ of Zuckerkandl was managed on single-agent thalidomide for over 15 years with initial partial response and now stability of disease. The question remains as to whether his response is due to his treatment, disease biology, or a combination of both.
